# A Comprehensive Study of Cutaneous Fibropapillomatosis in Free-Ranging Roe Deer (*Capreolus capreolus*) and Red Deer (*Cervus elaphus*): from Clinical Manifestations to Whole-Genome Sequencing of Papillomaviruses

**DOI:** 10.3390/v12091001

**Published:** 2020-09-08

**Authors:** Jernej Kmetec, Urška Kuhar, Aleksandra Grilc Fajfar, Diana Žele Vengušt, Gorazd Vengušt

**Affiliations:** 1 Institute of Pathology, Wild Animals, Fish and Bees, Veterinary Faculty, University of Ljubljana, Gerbičeva 60, 1000 Ljubljana, Slovenia; kmetec.jernej@gmail.com (J.K.); diana.zelevengust@vf.uni-lj.si (D.Ž.V.); 2 Institute of Microbiology and Parasitology, Veterinary Faculty, University of Ljubljana, Gerbičeva 60, 1000 Ljubljana, Slovenia; urska.kuhar@vf.uni-lj.si (U.K.); aleksandra.grilcfajfar@vf.uni-lj.si (A.G.F.)

**Keywords:** papillomavirus, fibropapillomatosis, red deer, roe deer, genome, NGS

## Abstract

Papillomaviruses (PVs) are an extremely large group of viruses that cause skin and mucosa infections in humans and various animals. In roe deer and red deer, most PVs belong to the *Deltapapillomavirus* genus and cause neoplastic changes that are generally described as fibropapillomas. Despite the wide distribution of roe and red deer throughout Europe and beyond, the data in the scientific literature regarding the widespread distribution of PVs and the genetic variability of PV genomes in these species are rather scarce. This study describes cutaneous fibropapillomatosis cases in roe and red deer with clinical manifestations that are typical of infections with PVs. In all cases, the presence of PV DNA was confirmed using PCR, followed by Sanger sequencing of the partial *L1* gene. The complete PV genomes were determined in all the investigated samples using next-generation sequencing technology, revealing infections of roe deer with the CcaPV1-type and red deer with the CePV1v-type variant. A comparison of the complete CcaPV1-type and CePV1v-type variant genome sequences reported here with already available complete genome sequences in GenBank revealed their great genetic stability across time and space.

## 1. Introduction

Papillomaviruses (PVs) are nonenveloped viruses with a circular double-stranded DNA genome that ranges in size between 6800 and 8400 bp [1,2]. PV genomes encode 8–10 proteins and can be divided into three functional regions: the long control region (LCR), the early region (E) and the late region (L). The capsid is composed of the L1 and L2 proteins. The E1 and E2 proteins are involved in replication and transcription. The E5, E6 and E7 proteins induce cellular DNA replication. The E4 protein may represent a late-functioning protein and binds to specific cytoskeleton structures [3,4].

PVs are an extremely large group of viruses, with more than 400 human and animal PV reference genomes listed in the curated Papillomavirus Episteme (PaVE) database (http://pave.niaid.nih.gov). Almost half of these genomes are of animal origin, including those of viruses from cervid species. Most of these viruses are species-specific [5,6], but cross-infections have also been reported [5,7,8,9]. It was reported that some animal species are often infected by PVs belonging to different taxonomic groups [10,11,12]. Cross-infections with *Deltapapillomavirus* BPV-1, BPV-2 and CePV-1 have been identified in the healthy skin of wild ruminants, and cross-infections with BPVs from the genus *Xipapillomavirus* have been identified, both as single infections and in association with *Deltapapillomavirus* BPV-1 and BPV-2 [13,14]. Coinfections with BPV-1 and CePV-1 have been reported in red deer with fibropapillomatosis [11,14]. These findings suggest that wild ruminants are a possible reservoir of PVs, which can affect domestic ruminants as well [11,13].

PVs are responsible for skin and mucosa infections of humans and various animals, in which they can appear asymptomatic or cause different neoplastic changes, ranging from self-limited warts to cancers [5,10,12,13]. In cervids, most of the detected PVs belong to the *Deltapapillomavirus* genus and cause neoplastic changes described as fibromas, papillomas, fibropapillomas or warts [15]. In infected roe and red deer, the lesions appear as multiple benign tumours in the form of fibropapillomas, predominately on the neck, head, abdomen and extremities. Larger tumours can become ulcerated and eroded. In rare cases, tumours form metastases in the lungs [16,17]. Although it is still not known exactly how PV infections are transmitted in nature among wild cervid populations, epidemiological studies suggest the transmission of PVs by direct cutaneous or mucosal contact, contaminated vegetation and/or haematophagous insects [15,18,19].

PV infections of roe deer appear as endemic infections in Hungary, Austria, Croatia [16] and Slovakia [15]. The spreading of fibropapillomatosis in roe deer was reported in all countries where the disease occurs [15,16]. The only characterisation of the complete genome of the CcaPV-1 type belonging to the *Deltapapillomavirus* genus was carried out in Hungary by Erdélyi et al. [20], who were the first to link the virus to the development of fibropapillomatosis in roe deer. A sporadic occurrence of fibropapillomatosis in red and roe deer was reported in Spain, France, England, Austria [16,17], the Czech Republic [21], Portugal [11] and Italy [14]. Scagliarini et al. [14] were the first to publish the entire genome of a PV (CePV-1v) belonging to the *Deltapapillomavirus* genus in red deer with expressed clinical symptoms. Based on genomic analysis, CePV-1v was classified as a type variant of CcaPV-1. Recently, two additional complete CePV-1v genomes from red deer with fibropapillomatosis in Italy were sequenced and published [22], showing almost 100% nucleotide identity with the previously published CePV1v. A case of a red deer infected with CePV-2, which belongs to the genus *Epsilonpapillomavirus*, and associated with pigmented papillomas was reported in New Zealand [6].

The objective of the present study was to investigate fibropapillomatosis cases in roe and red deer in Slovenia through histopathology and virus diagnosis using PCR. Additional aims were to determine the nucleotide sequences of whole PV genomes and to compare them with each other and with other viral strains from other countries.

## 2. Materials and Methods

### 2.1. Samples

**Figure 1:**
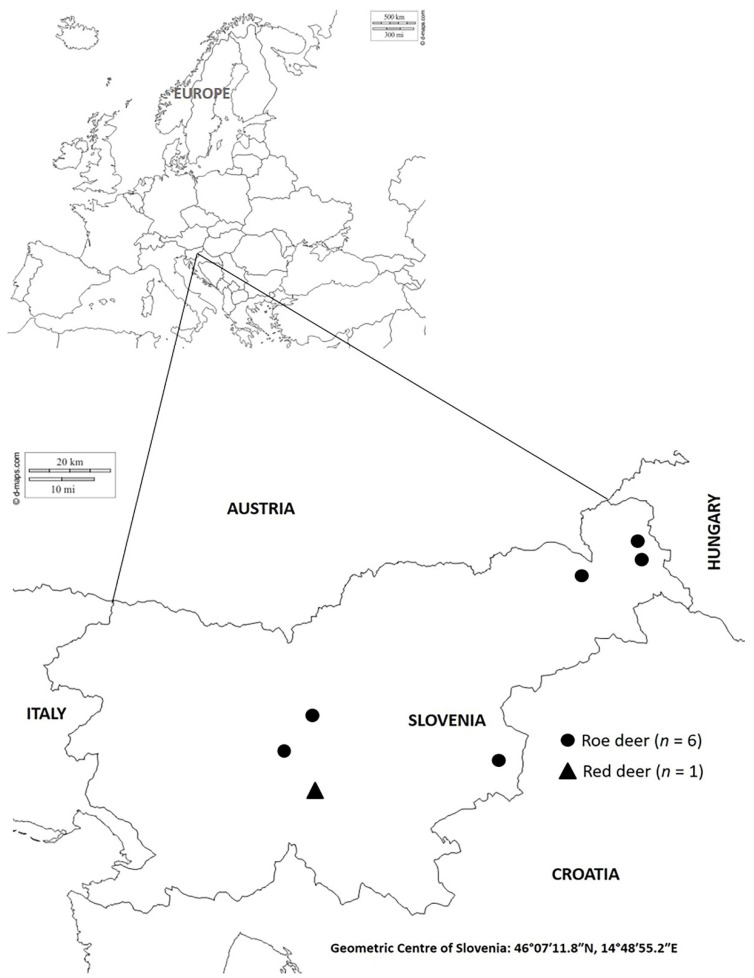
Geographical locations of the collection of roe and red deer samples from fibropapilloma tissues that tested positive for the presence of papillomavirus (PV).

Between 2014 and 2016, a total of seven free-range adult roe deer (*n* = 6) and red deer (*n* = 1) with clinical signs of fibropapillomatosis were harvested by hunters during either the regular annual cull or their emergency removal from the wild due to illness according to the hunter inspector’s decision (Figure 1). The animal species, age, sex and year of harvesting are presented in Table 1. Eruption patterns and tooth wear were used for the age estimation of the deer. Age was estimated subsequently by an authorised committee of hunters during an obligatory annual verification of hunted ungulates [23]. All roe deer carcasses and only the neoplastic change from red deer were submitted to the Institute for Pathology, Wild Animals, Fish and Bees, Veterinary Faculty, University of Ljubljana, Slovenia (Institute), for a necropsy, histopathologic examination and virus diagnosis with molecular techniques. No ethical/welfare authority approval was required, as all samples were collected postmortem.

### 2.2. Histopathology

Tissue samples of lesions were collected for histopathology. The samples were fixed in 10% buffered formalin, processed, embedded in paraffin, sectioned and stained with haematoxylin and eosin according to standard protocols.

### 2.3. PCR and Sanger Sequencing of the Partial L1 Gene

The tissue samples collected during the postmortem examinations were stored at −70 °C pending molecular testing. To prepare the suspensions of the tissue samples, 1 cm^3^ of tissue was added to 9 mL of RPMI medium 1640 (Thermo Fisher Scientific, Carlsbad, CA, USA). The suspensions were homogenised and centrifuged at 2000× *g* for 10 min, and the supernatant was stored at −70 °C if not immediately processed. The supernatant was employed for nucleic acid extraction using the DNeasy Blood & Tissue Kit (Qiagen, Hilden, Germany) according to the manufacturer’s instructions. PCR with a combination of primers (CanPVf: 5′-CTTCCTGAWCCTAAYMAKTTTGC-3′, FAP64: 5′-CCWATATCWVHCATITCICCATC-3′), which were already described by Lange et al. [24], amplified a 383 bp long fragment of the *L1* gene that was used for the detection of PVs [24,25]. The PCR products were subjected to electrophoresis in a 1.8% agarose gel, and the positive PCR products were purified and sequenced using the Sanger method (Macrogen Europe B.V., Amsterdam, Netherlands) according to the expected size of the DNA fragments. The obtained nucleotide sequences were assembled and edited using SeqMan and EditSeq implemented in the DNASTAR program (Lasergene, Madison, WI, USA) and compared with the sequences published in GenBank (National Center for Biotechnology Information, NCBI) using BLASTn (NCBI).

### 2.4. Whole-Genome Sequencing

For whole-genome sequencing with next-generation sequencing (NGS) technology, a Covaris M220 focused ultrasonicator (Covaris, Woburn, MA, USA) was used to fragment the extracted DNA, targeting peak fragment lengths of 400 bp. The fragmented DNA was purified and concentrated with magnetic Agencourt AMPure XP Beads (Beckman Coulter, Beverly, MA, USA) and used for barcoded NGS library preparation with the GeneRead™ DNA Library L Prep Kit (Qiagen, Hilden, Germany) according to the manufacturer’s instructions. Agencourt AMPure XP Beads (Beckman Coulter, Beverly, MA, USA) were used for the purification and double size selection of the NGS library fragments. The NGS library concentration was determined using a QIAseq Library Quant Assay Kit (Qiagen, Hilden, Germany) and a Qubit v.3.0 fluorometer (Thermo Fisher Scientific, Waltham, MA, USA). The emulsion PCR and enrichment were carried out using the Ion PGM™ Hi-Q™ View OT2 Kit reagents (ThermoFisher Scientific–Ion Torrent, Carlsbad, CA, USA) according to the manufacturer’s instructions. The sequencing of the NGS library was performed on the Ion PGM platform using the Ion PGM™ Hi-Q™ View Sequencing Kit reagents (ThermoFisher Scientific–Ion Torrent, Carlsbad, CA, USA).

### 2.5. Bioinformatic Analysis of NGS Data

The sequenced reads were quality checked and trimmed using the Ion Torrent Suite v.5.6.0 (ThermoFisher Scientific–Ion Torrent, Carlsbad, CA, USA). For the genome assembly, the reads were mapped to the reference genomes (accession numbers NC_011051 and JQ744282) using the Geneious software suite v.11.0.5 (Biomatters Ltd., Auckland, New Zealand). The Geneious software suite v.11.0.5 was also used for further downstream bioinformatic analyses of the assembled CePV1v and CcaPV1 genomes. The consensus sequences were aligned with the reference genome CcaPV1 (NC_011051) and the CePV1v genomes (JQ744282 and MN985322) using MAFFT [26]. The open reading frames (ORFs) were predicted with the Geneious ORF Finder, and the assembled genomes were annotated based on multiple alignments relative to the CePV1v and CcaPV1 genomes. The sequences were deposited in GenBank under the following accession numbers: MT755964 (CePV1v strain 64-14), MT774138 (CcaPV1 strain 31-16), MT774139 (CcaPV1 strain 32-16), MT774140 (CcaPV1 strain 37-14), MT774141 (CcaPV1 strain 63-15), MT774142 (CcaPV1 strain 103-16) and MT774143 (CcaPV1 strain 84-14).

### 2.6. Phylogenetic Analysis

*Deltapapillomavirus* genomes were retrieved from GenBank and used in the phylogenetic comparisons. Human papillomavirus (HPV) was used as an outgroup sequence. Multiple alignments of the concatenated E1, E2, L1 and L2 gene sequences were generated using MAFFT [26]. A phylogenetic tree was constructed with MEGA v.7.0.21 [27] using the Neighbor-Joining NJ method with the Tamura 3 substitution model with the gamma parameter (T92+G). Statistical support for the phylogenetic tree was evaluated via bootstrapping based on 1000 repetitions.

### 2.7. Data Availability Statement

The authors confirm that the data supporting the findings of this study are available within the article and are openly available in the GenBank database at https://www.ncbi.nlm.nih.gov/nucleotide/ under accession numbers MT774138-43 and MT755964.

## 3. Results

### 3.1. Gross Pathology and Histopathology

**Figure 2:**
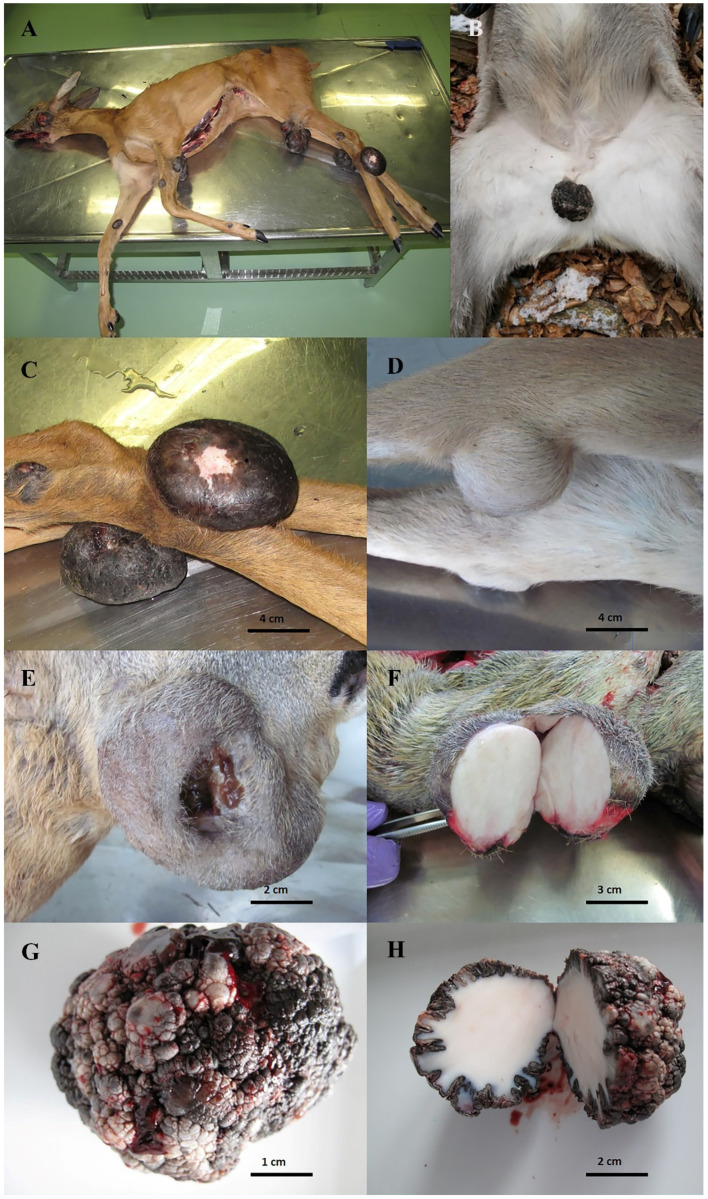
(**A**) Macroscopic lesions of fibropapillomas of various sizes localised on the head and extremities of an infected roe deer. (**B**) Macroscopic lesion of solitary fibropapilloma of an infected red deer in the groin area. (**C**) Pedunculated, round, firm, pigmented, eroded and hairless fibropapillomas of a roe deer. (**D**) Round, firm and hairy fibropapilloma of a roe deer. (**E**) Large, ulcerated fibropapilloma of a roe deer. (**F**) Cut surface of a roe deer fibropapilloma with a compact, shiny and white appearance of the firm connective tissue. (**G**) Fibropapilloma of a red deer showing a cauliflower-like mass and a hairless and pigmented surface. (**H**) Cut surface of a red deer fibropapilloma with a compact, shiny and white appearance of the firm connective tissue and a pronounced papillary structure of the external surface.

The postmortem investigations in roe deer showed firm, round, usually hairless and pigmented, eroded or ulcerated and, in all cases, multiple skin tumours of varying size and number (Figure 2). They were located on the head, neck and legs of the infected animals. The tumour specimens ranged from 4 to 16 in number and from 1 to 14 cm in size. In the red deer, the tumour was solitary, located in the groin area and consisted of a rough, pigmented, hairless and cauliflower-like mass. The cut surface of all the tumours revealed a compact, shiny mass with a white centre. Histopathological examinations revealed elements that were characteristic of neoplastic lesions caused by PV, including orthokeratotic hyperkeratosis, hyperpigmentation, connective tissue, fibroblast proliferation and keratohyaline granules in the cytoplasm of enlarged keratinocytes. The mitotic index was low, with less than one mitosis per high power field on average. There was no evidence of blood or lymph vessel invasion or tumour metastasis. The above-described characteristics observed in this study suggest the benign nature of the diagnosed tumours. The neoplastic changes in all the investigated animals were diagnosed as cutaneous fibropapillomas based on gross pathology and histopathology.

### 3.2. PCR and Sanger Sequencing of the Partial L1 Gene

All seven samples from the fibropapilloma tissues tested positive for the presence of PV according to the PCR amplification of the *L1* gene fragment. The PCR products were subjected to Sanger sequencing, which, according to BLASTn searches, showed an almost 100% nucleotide sequence identity of the roe deer PVs with the CcaPV1 (NC_011051) sequence and a 100% nucleotide sequence identity of red deer PVs with CePV1v (JQ744282 and MN985322) sequences. Based on the partial *L1* gene sequences, the roe and red deer PVs investigated in this study belong to the *Deltapapillomavirus* 5 species, with the roe deer PVs belonging to the CcaPV1 type and the red deer PV belonging to CePV1v, which is a CcaPV1-type variant.

### 3.3. Whole-Genome Sequencing and Genome Analysis

**Figure 3:**
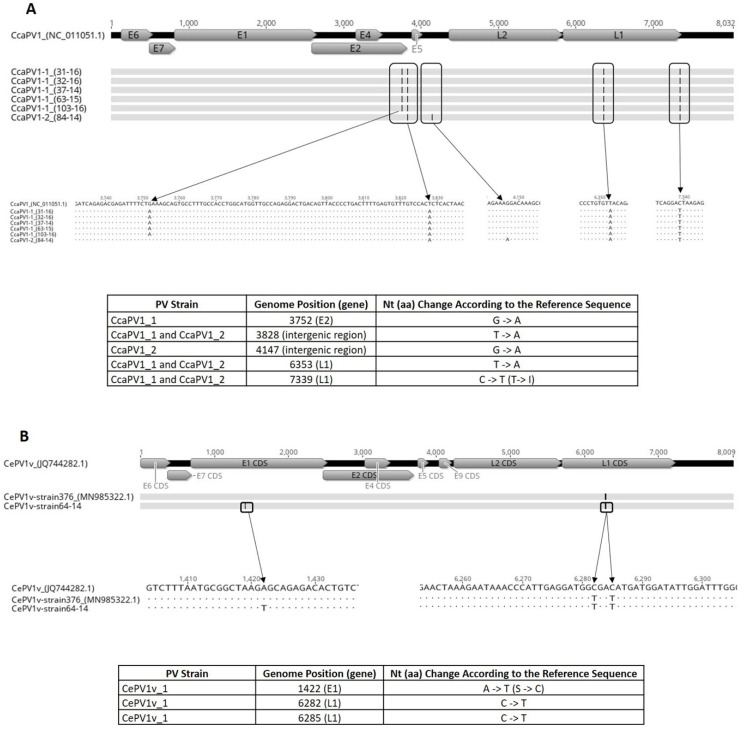
Multiple alignments of roe (**A**) and red deer (**B**) PV genomes from this study compared to the reference CcaPV1 genome and CePV1v genomes, respectively, highlighting the distribution of nucleotide substitutions, which are described in detail in the tables. Nt: nucleotide, aa: amino acid.

To characterise the roe deer and red deer PVs investigated in this study in detail, their complete genomes were determined using NGS technology. The NGS results and genome assembly results obtained using the reference mapping approach are presented in Table 2. The whole-genome analysis of the roe and red deer PVs investigated in this study confirmed their taxonomic classification in the *Deltapapillomavirus* 5 species suggested by the BLASTn analysis of the partial *L1* gene sequence, showing that the roe deer PVs belonged to the CcaPV1 type and that the red deer PV belonged to the CePV1v type. The analysis of multiple alignments showed that five roe deer PV genomes (CcaPV1-1; 31-16, 32-16, 103-16, 63-15 and 37-14) were 100% identical and differed from the reference CcaPV1 PV genome by four nucleotide substitutions (99.95% nucleotide identity). Three of the nucleotide substitutions were located within coding regions within the *E2* and *L1* genes, with one within the *E2* gene and two within the *L1* gene. Two nucleotide substitutions, one in the *E2* gene and one in the *L1* gene at genome position 6353, were synonymous and one nucleotide substitution in the *L1* gene at genome position 7339 was non-synonymous. One roe deer PV genome (CcaPV1-2 (84-14)) differed from the other five roe deer CcaPV1-1 genomes by two nucleotide substitutions, one of which was located in a coding region within the *E2* gene, resulting in a synonymous substitution. Compared to the reference CcaPV1 genome, the CcaPV1-2 (84-14) genome differed by four nucleotide substitutions (99.95% nucleotide identity), two of which were within a coding region (both within the *L1* gene). One nucleotide substitution at genome position 6353 was synonymous and one nucleotide substitution in the *L1* gene at genome position 7339 was non-synonymous (Figure 3a). In the *L1* gene nucleotide sequence, all six roe deer PVs differed from the reference CcaPV1 genome by 0.13%. The red deer CePV1v PV genome (64-14) was aligned with the CePV1v genomes (JQ744282 and MN985322). The analysis of multiple alignments showed that the CePV1v (64-14) genome differed from the reference CePV1v genome (JQ744282) by three nucleotide substitutions (99.96% nucleotide identity). The nucleotide substitutions were within coding regions within the E1 and *L1* genes, with one non-synonymous nucleotide substitution in the *E1* gene at genome position 1422 and with two synonymous nucleotide substitutions in the *L1* gene at genome positions 6282 and 6285 (Figure 3b). In the *L1* gene nucleotide sequence, the red deer PV differed from the reference CePV1v genome (JQ744282) by 0.13%. The comparison of the CePV1v (64-14) genome to CePV1v_strain376 (MN985322) showed a difference of one non-synonymous nucleotide substitution (99.99% nucleotide identity) in the *E1* gene (Figure 3b).

### 3.4. Phylogenetic Analysis

**Figure 4:**
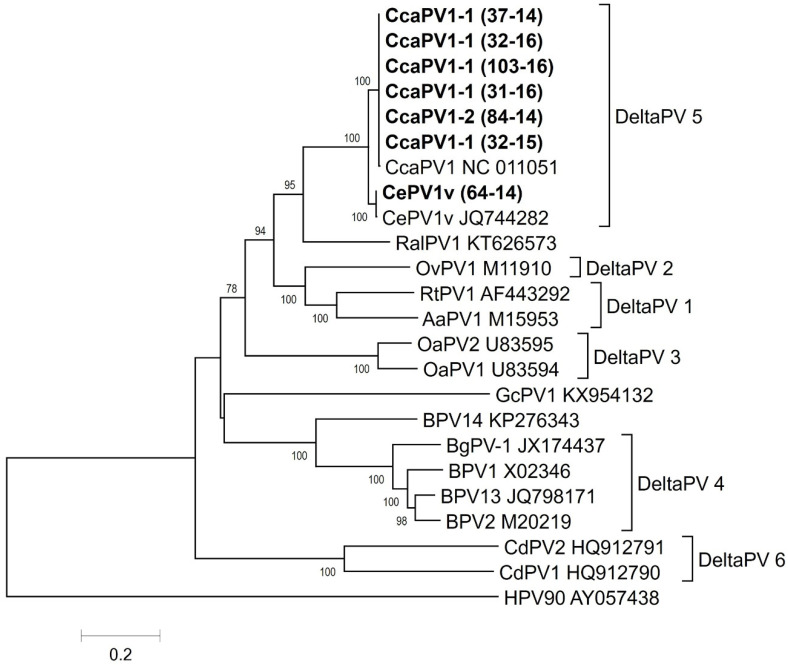
Neighbor-Joining (NJ) phylogenetic tree with the (T92+G) substitution model of the concatenated *E1*, *E2*, *L1* and *L2* gene sequences. Statistical support for the phylogenetic tree was evaluated via bootstrapping based on 1000 repetitions. Bootstrap values lower than 70 are not shown. The Slovenian PV strains are indicated in bold.

The phylogenetic analysis confirmed that the roe and red deer PVs investigated in this study belonged to the *Deltapapillomavirus* 5 species. Specifically, the roe deer PVs belonged to the CcaPV1 type and the red deer PV belonged to the CePV1v-type variant (Figure 4).

## 4. Discussion

Roe deer can be found throughout most of Europe, including western Russia [28], and in some countries outside of Europe [29]. The red deer distribution extends from Europe into North Africa and the Middle East [29,30,31,32]. Despite the wide distribution of these species throughout the continent and beyond, the data available in the scientific literature regarding the widespread nature of PVs and the genetic variability of PV genomes in these species are rather scarce. In Europe, fibropapillomatosis in wild cervids was first described in Hungary in the early 1960s by Kocsner [33]. The only characterisation of the complete genome of the roe deer CcaPV-1 type belonging to the *Deltapapillomavirus* genus was published by Erdélyi et al. [20]. Erdélyi [34] described the endemic distribution of roe deer fibropapillomatosis, indicating a low prevalence that was limited to the Pannonian Basin. Later, fibropapillomatosis in roe deer was clinically and pathohistologically identified in Serbia [35] and Croatia [36], while in the Czech Republic [21] and Slovakia [15], PV infection was confirmed using PCR. In red deer, fibropapillomatosis appears to be occurring sporadically throughout Europe and has thus far been described in Italy, Spain, France, England, Austria, Hungary and Portugal [11,14,22,34]. The first characterisation of the entire genome of PV (CePV-1v) belonging to the *Deltapapillomavirus* genus in red deer was described by Scagliarini et al. [14]. Recently, two complete CePV-1v genomes, which were 100% identical, were described in red deer from Italy by Gallina et al. [22]. Here, we describe fibropapillomatosis cases in roe deer (*n* = 6) and red deer (*n* = 1), including the complete genome sequencing of PVs from all of the investigated cases that have been sent to our institute for diagnostic investigation since 2014. In this study, the neoplastic changes of seven deer were investigated and diagnosed as cutaneous fibropapillomas based on gross pathology and histopathology. The PV DNA was detected in the tissue samples of fibropapillomas in all seven cases using PCR. The neoplastic lesion size and numbers, as well as the gross pathology and histopathology of the neoplastic lesions observed in this study, were consistent with previous studies of roe deer and red deer cutaneous fibropapillomatosis [14,15,16,17,22,34]. Thus, it can be assumed that the firm, round, usually hairless and pigmented, eroded or ulcerated lesions located in almost all body parts are characteristic for cutaneous fibropapillomatosis associated with the CcaPV1 infections in roe deer. Meanwhile, in red deer, the lesions, characteristic for cutaneous fibropapillomatosis associated with the CePV1v infections, appeared rough (with a cauliflower-like appearance), pigmented and hairless and were located in the inner hind leg or the abdominal region. According to previous reports [15,16,17,21], older roe deer appear to be more prone to disease manifestation than younger animals, while in red deer, fibropapillomas are mostly found in young animals. A similar pattern was observed in this study.

Histopathologic reports of archived samples sent by Slovenian hunters describing fibropapillomatosis in red and roe deer suggest that the disease may be present in some parts of eastern Slovenia, at least since 1996. As the first clinical cases were observed near the Slovenia–Hungary border, the infection was likely introduced to Slovenia from Hungary. In 2014 and 2016, the first cases of PV infection in Slovenia in roe and red deer, respectively, were confirmed using PCR diagnosis. Previously, the disease was diagnosed in roe deer using gross pathology and histopathology.

The abundance or density of the roe deer population is higher in the lowland areas of central and eastern Slovenia [37]. All of the detected cases of fibropapillomatosis in Slovenia came from these areas, similar to the observations of Rajský et al. [15], who reported that fibropapillomatosis cases were more common in hunting grounds with a high density of roe deer. PV infections in roe deer in Slovenia occur sporadically rather than being endemic, as is typical in Hungary [16] or Slovakia [15], where the expansion of roe deer fibropapillomatosis has occurred. To date, only one case of fibropapillomatosis in red deer has been clinically and molecularly confirmed in Slovenia. Viral fibropapillomas in red deer have been sporadically described in different European countries [11,14,17]. According to the present data, the disease is spreading very slowly from the eastern part to the central part of Slovenia; in the southwestern part of Slovenia, the disease has not yet been noted. The estimated population sizes of roe deer and red deer in Slovenia are 110,000 and 20,000, respectively [38]. According to the population size and the numbers of clinically, pathohistologically and, later, PCR-confirmed cases, we can assume that the prevalence of PV infections in roe deer and red deer in Slovenia is low. This is consistent with most reports from other European countries [16,17,21,33,35,36].

PVs exhibit a slow but astonishing evolutionary success, and traditional assumptions about the mechanisms of PV evolution include that they have very low mutation rates, co-diverge with their hosts and are host-specific [3,39,40,41]. However, various alternative mechanisms that drive PV evolution have recently been proposed, such as cross-species transmission, recombination and incongruence in the phylogenetic trees of viruses and their hosts [3,40,42,43,44]. The general conclusions about PV evolution may still be unclear and speculative, as knowledge about nonhuman PV diversity is particularly unbalanced and limited [3,42,45]. The bovine BPV-1 and BPV-2 types that infect cattle provide an example of cross-species transmission, not only between closely related hosts but also between more distantly related species, as they also infect buffaloes, bison, yaks, giraffes, tapirs, sable antelope, horses, donkeys and red deer [11,12,14,46]. The assumption that virus–host co-divergence alone was the driving force for current PV diversity has also been opposed by several studies describing distantly related PVs infecting the same host species, leading to incongruence in the phylogenetic trees of the viruses and their hosts [3,42,43,44]. In this study, very low variability between the CcaPV1-type genomes (99.95% identity at the complete genome level compared to the sequences deposited in GenBank) and the CePV1v-type variant genomes (99.96–99.99% identity at the complete genome level compared to the sequences deposited in GenBank) was observed, with five out of six CcaPV1 genomes being 100% identical. Similar results were observed by Gallina et al. [22], who reported two complete CePV1v genomes that were 100% identical. This observation is in agreement with the generally accepted evolutionary mechanism of PVs, which present a very slow mutation rate. In Slovenia, roe deer are the most widespread cervid species; they inhabit areas from the seacoast to the upper tree line. They can be found in forests and agrarian landscapes and have also adapted to live in open fields [47]. Red deer inhabit significantly smaller and more spatially isolated areas, as well as non-forested areas, such as meadows and agricultural fields, which are among their key feeding grounds [37]. Because these wild cervids share habitats with livestock, the transmission of infectious agents, including PVs, is possible. Thus, we can also expect infections with BPV types in the investigated wild cervids. However, in all the investigated cases of fibropapillomatosis, no coinfection with other PV types was detected, which is consistent with the traditional host specificity view of PV evolution mechanisms.

Region *L1* is used for phylogenetic and taxonomic analysis [39]. The taxonomic status of PV types, subtypes and variants is based on the traditional criterion that the sequences of their *L1* genes should be at least 10%, 2–10%, or maximally 2% dissimilar from one another. A new PV isolate is recognized as such if the DNA sequence of the L1 ORF differs by more than 10% from the closest known PV type when the complete genome is cloned. Differences between 2 and 10% homology define a subtype, and differences of less than 2% define a variant. The traditional PV types within a species share between 71 and 89% nucleotide identity within the complete L1 ORF [41]. Most of the effort applied to understand the evolution of PVs and their classification has been focused on HPVs and PVs in domestic animals, such as BPV types [10]. There are limited studies available concerning PVs from wildlife hosts [42,45], representing a gap in knowledge regarding our understanding of the evolution and diversification of PVs in the wild [10]. We found low variability (0.13%) of roe deer PV L1 ORF sequences in comparison to sequences available in GenBank, which according to de Villiers et al. [41], fall within the <2% sequence diversity category. Therefore, we could classify the sequences as CcaPV1 variant lineages or sublineages. Similar results were observed for the red deer PV L1 ORF sequence, which could also be classified as a variant lineage or sublineage, though belonging to the CePV1v type.

## 5. Conclusions

The study describes fibropapillomatosis cases in roe deer and red deer with clinical manifestations that are typical of infection with PVs. In all cases, the presence of PV DNA was confirmed using PCR, followed by Sanger sequencing of the partial *L1* gene. The complete PV genomes were determined in all of the investigated samples using NGS, revealing infections of roe deer with the CcaPV1-type and red deer with the CePV1v-type variant. A comparison of the complete CcaPV1-type and CePV1v-type variant genome sequences reported here to already available complete genome sequences available in GenBank revealed their great genetic stability over time and space.

## Figures and Tables

**Table 1:** Data of the investigated animals.

Animal Species/Year/Month of Harvesting	Sex	Age (in Years)
Roe deer/2014/August	Male	2
Roe deer/2014/December	Male	3
Roe deer/2015/October	Female	6
Roe deer/2016/May	Male	6
Roe deer/2016/May	Male	3
Roe deer/2016/December	Female	6
Red deer/2016/September	Female	1

**Table 2:** Results of the whole-genome sequencing of the PV genomes and the assembly statistics.

PV Strain	Total Reads	Mapped Reads	Mean Read Length
CcaPV1-1 (31-16)	197,354	312	326
CcaPV1-1 (32-16)	229,894	737	302
CcaPV1-1 (103-16)	206,940	1519	279
CcaPV1-1 (63-15)	213,479	241	309
CcaPV1-1 (37-14)	254,725	380	292
CcaPV1-2 (84-14)	317,345	636	278
CePV1v-1 (64-16)	2,867,278	10,010	282
